# Identification of miRNAs and their target genes associated with improved maize seed vigor induced by gibberellin

**DOI:** 10.3389/fpls.2022.1008872

**Published:** 2022-09-13

**Authors:** Yunqian Jin, Bin Wang, Lei Tian, Linxi Zhao, Shulei Guo, Hengchao Zhang, Lengrui Xu, Zanping Han

**Affiliations:** ^1^College of Agronomy, Henan University of Science and Technology, Luoyang, China; ^2^State Key Laboratory of Cotton Biology / Institute of Cotton Research of Chinese Academy of Agricultural Sciences / School of Agricultural Sciences, Zhengzhou University, Henan, Zhengzhou, China / Key Laboratory for Cotton Genetic Improvement, MOA, Anyang, Henan, China; ^3^College of Agronomy, Henan Agricultural University, Zhengzhou, China; ^4^Cereal Institute, Henan Academy of Agricultural Science/Henan Provincial Key Laboratory of Maize Biology, Zhengzhou, China

**Keywords:** maize, seed vigor, miRNAs, gibberellin, degradome sequencing, glycan degradation and galactose metabolism

## Abstract

High seed vigor is crucial for agricultural production owing to its potential in high quality and yield of crops and a better understanding of the molecular mechanism associated with maize seed vigor is highly necessary. To better understand the involvement and regulatory mechanism of miRNAs correlated with maize seed vigor, small RNAs and degradome sequencing of two inbred lines Yu537A and Yu82 were performed. A total of 791 mature miRNAs were obtained with different expressions, among of which 505 miRNAs were newly identified and the rest miRNAs have been reported before by comparing the miRNAs with the sequences in miRbase database. Analysis of miRNA families showed maize seeds contain fewer miRNA families and larger miRNA families compared with animals, indicating that functions of miRNAs in maize seeds were more synergistic than animals. Degradome sequencing was used to identify the targets of miRNAs and the results showed a total of 6,196 targets were obtained. Function analysis of differentially expressed miRNAs and targets showed Glycan degradation and galactose metabolism were closely correlated with improved maize seed vigor. These findings provide valuable information to understand the involvement of miRNAs with maize seed vigor and these putative genes will be valuable resources for improving the seed vigor in future maize breeding.

## Introduction

Maize, one of the most popular crops all over the world, plays an important role in overall agricultural production. Maize seed vigor is a kind of comprehensive seed characteristic, including germination rate, germination potential, seedling growth potential, plant resistance and productivity potential, etc., which determines the ability of rapid and healthy germination in the complex field environment and effects crop quality and yield (Zhang et al., 2009; Dang et al., 2014). Therefore, it is an important task to study the molecular mechanism of maize seed vigor and to create high-vigor maize germplasm.

It was suggested that gibberellins (GA) may play an important role in maturity germination. In some species, bioactive GAs are thought to be present and apparently important during early embryogenesis and germination of mature seeds (White et al., 2000). Previous research reported that plant hormones within seeds, including gibberellins (GA), abscisic acid (ABA), auxin and ethylene, all contribute to the balance of plant hormones and germination performance (Kucera et al., 2005; Linkies and Leubner-Metzger, 2012; Miransari and Smith, 2014). However, although much effort has been done, it is urgent to study the key genes and signaling pathways associated with gibberellin-induced improvement of maize seed vigor.

Many documents reported that microRNAs (miRNAs) infers to those endogenous, small, singe-stranded non-coding RNAs with the length of usually from 18 to 25 nt, which play an important role at the transcriptional and posttranscriptional regulation level in regulation of seed vigor in maize, rice and many other plants (Bartel, 2004; Zamore and Haley, 2005; Zuo et al., 2018; Huang et al., 2020). Some miRNAs derive from characterized genes with specific regulation functions, and some are from the introns of protein-coding genes or some other noncoding genomic regions, such as retrotransposons (Axtell and Bowman, 2008; Cuperus et al., 2011). MiRNAs are usually transcribed from larger precursors that contain a stem-loop structure, known as pri-miRNAs, and then pre-miRNAs are eventually processed into mature miRNAs by dicer-like (DCL) proteins with the assistance of some other binding proteins (Park et al., 2002; Xie et al., 2005; Jia et al., 2014). Finally mature miRNAs combined with the RNA induced silencing complex (RISC), which would lead the mature single miRNAs to guide the RNA slicing activity of AGO1 to recognize their targets through perfect or near-perfect complementarity to regulate the expression of target genes, which was a bit different from miRNAs in animals (Bonnet et al., 2006; Mallory and Vaucheret, 2006; Voinnet, 2009; Khan et al., 2014).

Recently, increasing number of miRNAs related with plant seeds have been identified and functionally studied in plants, such as *Arabidopsis* (Mehdi et al., 2021), rice (Parmar et al., 2020), maize (Zhao et al., 2022) and *Brassica napus* (Jiang et al., 2021). MiR159, targeting two abscisic acid (ABA)-positive regulators MYB33 and MYB101, was proved to play a vital role in the seed germination process (Reyes and Chua, 2007). A deep-sequencing technique was used to decipher the molecular mechanism of miRNA-dependent gene regulation for heterosis of maize, finally 34 miRNAs belonged to 20 miRNA families were obtained (Ding et al., 2012). In rice, miR168 and miR817 were functionally identified in artificially aged seeds, indicating that they were closely associated with the rice seed vigor. MiR169 was high conserved in plants and it regulates the expression of those genes encoding the universal transcription factor subunit NUCLEAR FACTOR-Y subunit A (NF-YA), which regulates gene expression by binding the CCAAT box sequence in target promoters in responses to abiotic stress (Luan et al., 2015). Published document reported that one *ARF* gene and three DNA-binding transcription factor genes were targeted by non-conserved ta-siRNAs in maize ear development (Liu et al., 2014). Two targets *Gh_A12G1620* and *Gh_D01G0190* were proved to enhance the drought tolerance of transgenic *Arabidopsis thaliana* plants significantly (Lu et al., 2019). However, the molecular mechanism of differentially expressed miRNAs during the seed germination process in maize was still unclear. In this study, two maize inbred lines Yu82 and Yu537A, were selected for small RNAs and degradome sequencing to identify key miRNAs and target genes associated with seed vigor, which could provide valuable information for maize seed germination.

## Materials and methods

### Plant materials, seed germination and GA3 treatments

Two maize inbred lines, Yu82 and Yu537A were selected and used in this study. These two inbred lines were derived from Yuzong5, which is an improved population cultivar and is cultivated widely in China. Two inbred lines were planted in Sanya (China, E109°35′, N18°29′) with the same filed management conditions in 2016. The seeds were harvested at the same developmental stage (mature stage, black layer formed). Then these seeds with consistent physiological state were selected and surface-sterilized with 75% ethanol. The seeds of Yu537A were pretreated with 400 mg/L gibberellin (GA3) for its lower seed vigor. Two inbred lines were germinated on the two layers of filter paper with the moisture in sterile petri dishes (the diameter was about 12 cm). The seeds were incubated at a constant temperature of 25°C under a 14/10 h (light/dark) photoperiod with photosynthetically active radiation of 25 μmol photons m^−2^ s^−1^. Three replicates were used for each inbred line. All sample names were recorded and showed in Supplementary Table 1.

### RNA isolation

The embryos of five individual seeds were dissected with a sterile knife in each sample and then the samples were immediately frozen in liquid nitrogen and stored at −80°C. Total RNA was extracted using RNAprep Pure Plant Kit (Tiangen) following the procedure of the manufacturer. The concentration was measured by Nanodrop 2000 and integrity of total RNA was tested on 1.0% agarose gel.

### Small RNA library construction, sequencing and raw data analysis

Small RNA library construction was conducted with TruSeq Small RNA Sample Prep Kits (Illumina, San Diego, CA, United States). Small RNA libraries were sequenced with Illumina Hiseq2000/2500 and the sequencing read length is single-ended 1 × 50 bp. The program ACGT101-miR (LC Sciences, Houston, TX, United States) used for raw data analysis of miRNAs sequencing was independently developed. The main steps are below: (1) removal of 3′ adaptor and junk sequences to obtain clean sequences; (2) screening of miRNA length of 18–25 nt; (3) compared the sequencing data to other database, including Rfam and Repbase; Rfam is a family database of non-coding RNAs (NcRNAs), including rRNA, tRNA, snoRNA, snRNA, miRNA and other non-coding Rnas. We selected Rfam database to annotate the small RNA sequences obtained by sequencing. rRNA, snoRNA, snRNA, tRNA and other non-mirNA sequences were found and removed as far as possible; (4) identification of miRNAs by comparing the precursors to the genome; (5) differential expression analysis of miRNAs; and (6) target prediction of miRNAs.

### Degradome library construction and data analysis

The degradome cDNA library was constructed on the basis of the method (Axtell, 2013) described previously combined with Beads screening. Main steps are below: (a) mRNA 3′ and 5′ adaptor were captured by magnetic beads; (b) Mixed reverse transcription of biotinylated random primers and mRNA; and (c) PCR amplification. Completed degradome library was sequenced with Illumina Hiseq2000/2500.

Independently developed program ACGT101-DEG was used for degradome sequencing analysis and target genes prediction analysis was performed with CleaveLand (Addo-Quaye et al., 2009) program. Main steps are below: (a) The raw data obtained by sequencing is processed through a series of data analysis to obtain comparable sequencing sequences that can be used for subsequent analysis; (b) The degradation density files were generated by comparing the sequences of comparable pairs with cDNA databases of sequenced species; (c) Target gene prediction software Targetfinder was used to predict the target gene mRNA sequences paired with the small RNA sequences of the sequenced species; (d) Conjoint analysis was performed with target genes predicted and the degradation density files.

### Determination of α-hydrolase enzyme activity, β-hydrolase enzyme activity and soluble sugar content in maize seeds

Under the catalysis of α-hydrolase, starch could be converted to maltose, which could react with 3,5-dinitrosalicylic acid to produce brown 3-amino-5-nitrosalicylic acid. Then enzyme activity ofα-hydrolase were determinated according to its absorbance at the wave length of 410 nm. With the PNP β-G3 method, 1.0 g malt flour was used to extract β-hydrolase for 1 h. Then 0.2 ml PNP β-G3 and glucanase mixture were used as substrate, and the absorbance was determinated after the reaction for 10 min at the wave length of 410 nm. Anthrone colorimetric method was applied to determinate the soluble sugar content in maize seeds and the optimum conditions were as follows: the concentration of anthrone was 15 mg/mL^−1^, the optimum temperature for color reaction was 80°C, and heating time was 15 min. Under these conditions, an excellent linear relationship between the concentration of glucose and absorbance was obtained and the correlation coefficient was *R*^2^ = 0.9991. Based on this optimized method, a standard curve was performed (*R*^2^ = 0.9984), suggesting the standard curve has a significant linear relationship. All samples were determinated according to this method and the results showed good repeatability and an accuracy. Each sample contained three replicates.

## Results

### Effects of GA3 on the germination rate, germination potential and germination index of Yu537A seeds

**Figure 1:**
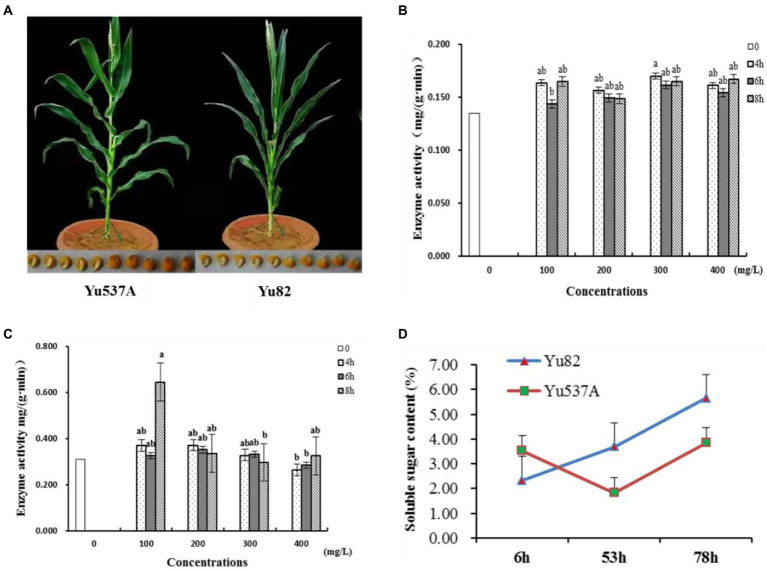
Analysis of enzyme activity of α-hydrolase and β-hydrolase, and soluble sugar content under different treatments of GA3 **(A)**, Phenotypes of two maize inbred lines Yu537A and Yu82. **(B)**, Enzyme activity analysis of α-hydrolase under different treatments of GA3. *X*-axis represents the concentrations of GA3 while *Y*-axis represents enzyme activity of α-hydrolase. Different columns present treatment time. **(C)**, Enzyme activity analysis of β-hydrolase under different treatments of GA3. *X*-axis represents the concentrations of GA3 while *Y*-axis represents enzyme activity of β-hydrolase. Different columns present treatment time. **(D)**, Soluble sugar content analysis of two maize inbred lines under different treatments of GA3. *X*-axis represents the treatment time with GA3 while *Y*-axis represents soluble sugar content in two maize inbred lines. Letters a and b represent the significant differences between different groups.

In this study, two maize inbred lines Yu82 with high germination rate and Yu537A with low germination rate were selected (Figure 1A; Table 1; Supplementary Table 2) to investigate the regulation mechanism in germination process. In order to explore the effects of GA3 on the germination of Yu537A seeds, standard germination tests were conducted according to the criterions of GB/T5520-2011 under the treatments of different GA concentrations (0, 100, 200, 300, and 400 mg/L) and durations (0, 4, 6, and 8 h). The results (Table 2) indicated the germination potential attained the highest value under the treatment of 400 ml/L GA3 for 8 h, which was extremely significantly higher than the other treatments. The highest germination rate was observed under the treatment of 200 ml/L GA3 for 8 h, which was significantly higher than CK. However, the highest germination index was observed under the treatment of 200 ml/L GA3 for 4 h, which was also significantly higher than CK. α-amylase and β-amylase were two key hydrolases in the germination process, and enzyme activity of two hydrolases were measured (Figures 1B,C). The results showed enzyme activity of α-amylase changed obviously, exhibiting the trend of first decrease and then increase. Compared with CK, the enzyme activity of α-amylase is higher than that in CK under different treatments of GA3. Moreover, enzyme activity of β-amylase attained the peak at the treatment of 100 mg/l for 4 h, significantly higher than CK. So it is speculated that higher enzyme activity of α-amylase and β-amylase induced by GA3 may be closely correlated with the germination of maize seeds. Besides, soluble sugar contents of two inbred lines were also determinated (Figure 1D) and the results indicated the soluble sugar content continuously increased in Yu82 seeds and accumulated to higher levels than those in Yu537A seeds except for the first several hours. In this study, it is suggested that enzyme activity α-amylase and β-amylase, and soluble sugar contents in two inbred lines significantly increased after GA3 treatment, indicating increased sugar content was very beneficial for the germination of maize seeds.

**Table 1:** Comparisons of germination between Yu82 and Yu537A.

Name of inbred lines	Germination rate (%)	Germination potential (%)	Germination index (%)
Yu82	90	85	15.13
Yu537A	60	60	4.14

**Table 2:** Effects of GA3 treatment with different concentrations and durations on the germination of Yu537A.

Treatment time (h)	Concentrations (mg/mL)	Germination potential (%)	Germination rate (%)	Germination index (%)
	CK	6.25	60.42	17.67
4	100	14.58^*^	65.63	18.36
	200	13.94	70.83	20.00^*^
	300	13.54	68.75	14.27
	400	9.78	73.96	15.53
6	100	6.25	85.42^*^	11.42
	200	13.42	84.38^*^	12.91
	300	17.36	80.21	11.43
	400	25.00^**^	89.58^*^	13.00
8	100	56.25^**^	80.21	12.71
	200	18.75^**^	90.58^*^	15.50
	300	43.75^**^	56.25	16.04
	400	36.25	59.38	15.90

* represents the significant difference at the 0.05% level while,

** represents the extremely significant difference at 0.01% level.

### Overview of the deep sequencing results

To investigate the possible miRNAs related with maize seed vigor, we profiled miRNA accumulation and differential expressions during seed germination. Deep sequencing generated a total number 1166415, 1063732, 634,029, 1,885,446, 1,560,914, and 870,636 unique reads from different libraries E_6h, S_6h, E_53h, S_53h, SGA_53h and S_78h, respectively (Table 3). Length distribution of valid reads is mainly 21, 22, and 24 bp, accounting for more than 87.60% of total valid reads. Repeat sequence category analysis of each sample showed most valid reads were derived from Gypsy, Copia, Sola, Ambal, Penelope and some other non-coding regions. Finally 791 mature miRNAs were obtained with different expressions, among of which 59 miRNAs were found with high expression, 324 miRNAs were found with low expression, while the rest of miRNAs were found with middle expression. In this study, 505 miRNAs were newly identified, 114 miRNAs were reported before and the rest were found to be slightly different with those already reported miRNAs by comparing the miRNAs sequences in miRbase database.

**Table 3:** Overview of sequenced reads.

Type	E_6h		S_6h		E_53h		S_53h		SGA_53h		S_78h	
	Unique	% of uniqiue	Unique	% of uniqiue	Unique	% of uniqiue	Unique	% of uniqiue	Unique	% of uniqiue	Unique	% of uniqiue
Raw reads	1,585,825	100.00	1,483,012	100.00	1,151,621	100.00	2,464,041	100.00	2,067,412	100.00	1,242,353	100.00
3ADT&length filter	405,277	25.71	408,746	29.90	507,499	44.41	562,242	33.38	489,949	23.72	362,173	34.40
Junk reads	8,125	0.51	6,002	0.40	4,945	0.43	9,835	0.34	9,243	0.45	5,120	0.41
Rfam	3,936	0.24	2,644	0.20	4,007	0.35	3,110	0.15	4,345	0.21	2,652	0.25
mRNA	2,162	0.14	1947	0.12	1,203	0.10	3,449	0.13	3,018	0.14	1813	0.14
Repeats	80	0.00	88	0.01	79	0.01	109	0.00	108	0.01	75	0.01
Valid reads	1,166,415	73.40	1,063,732	69.38	634,029	54.72	1,885,446	66.01	1,560,914	75.47	870,636	64.80
rRNA	2,209	0.02	1,267	0.01	2,953	0.03	1889	0.02	2,909	0.03	1794	0.02
tRNA	880	0.01	754	0.01	368	0	557	0.00	610	0.00	395	0
snoRNA	498	0	404	0	309	0	367	0	395	0	258	0
snRNA	112	0	71	0	74	0	130	0	159	0	31	0
Other Rfam RNA	236	0	148	0	303	0	166	0	271	0	175	0

### Identification of miRNA families

**Figure 2:**
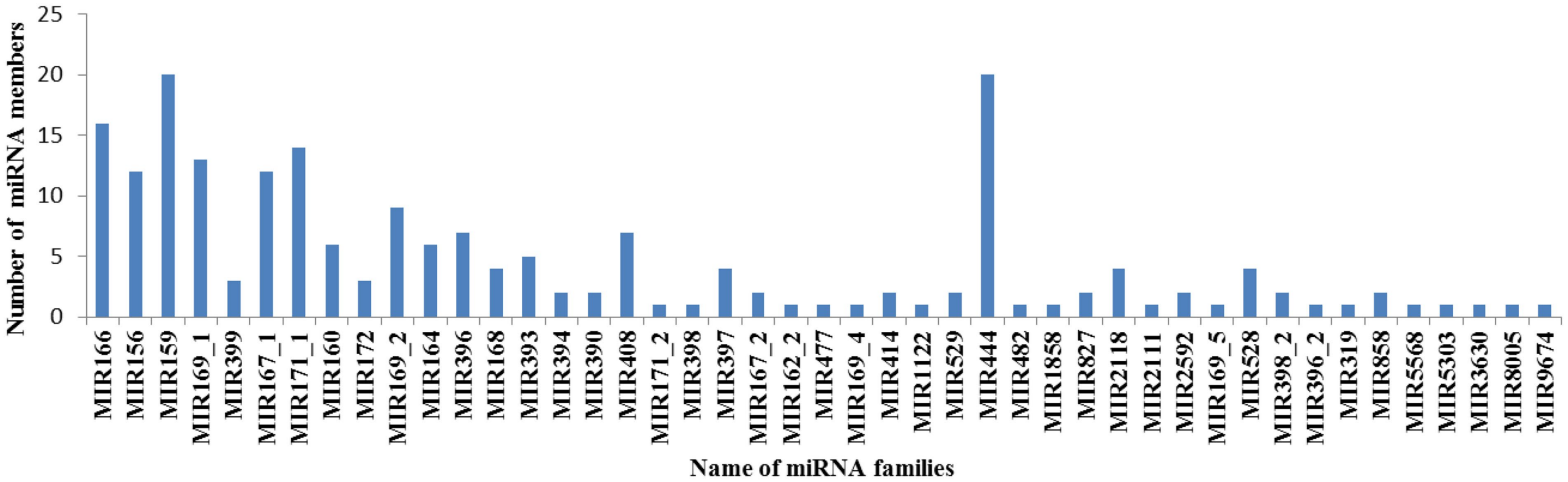
Distribution of miRNA members in each miRNA family *X*-axis represents the miRNA family name, while *Y*-axis represents the number of miRNA members in each miRNA family.

In plants, the members in each MiRNA family could reflect their conservation over a long period of evolution and regulation ability of their targets (Lu et al., 2019). All miRNAs obtained in this study could be divided into 45 miRNA families with the copy numbers varying from 1 to 20 (Figure 2; Supplementary Table 3). Previous research have reported that gain or loss of miRNAs is closely correlated with tandem gene duplication, whole-genome duplication and segmental duplication events in the plant genome (Li and Mao, 2007). In our research, approximately 17 miRNA families contained only 1 member, suggesting that these miRNAs were highly conserved. These evolutionarily conserved miRNAs were also found in other species, such as *Brassica oleracea* (Li et al., 2017), *Solanum tuberosum* (Qiao et al., 2017), *Glycine max* (Katara et al., 2010), and *Physcomitrella patens* (Wan et al., 2011). Among of all miRNA families, miR444 and MiR159 were two largest miRNA families, each containing 20 family members. This was consistent with previous researches. The average number of each family was approximately 4.53, which was a bit higher than that in previous reports in rice (Li and Mao, 2007), *Gossypium arboreum* (Farooq et al., 2017), and *Gossypium hirsutum* L. (Lu et al., 2019). Previous researches reported that miRNA families in animals usually contains less than 2.0 members, such as human contains 1.37, mouse contains 1.35, fruit fly contains 1.20 and chicken contains 1.76 members. As it is known to all, miRNA members of a miRNA family always perform similar functions (Lu et al., 2019). Compared with animals, maize seeds contain fewer miRNA families and larger miRNA families, so it is inferred that functions of miRNAs in maize seeds were more synergistic than animals.

### Identification of miRNAs putatively associated with improved maize seed vigor

**Figure 3:**
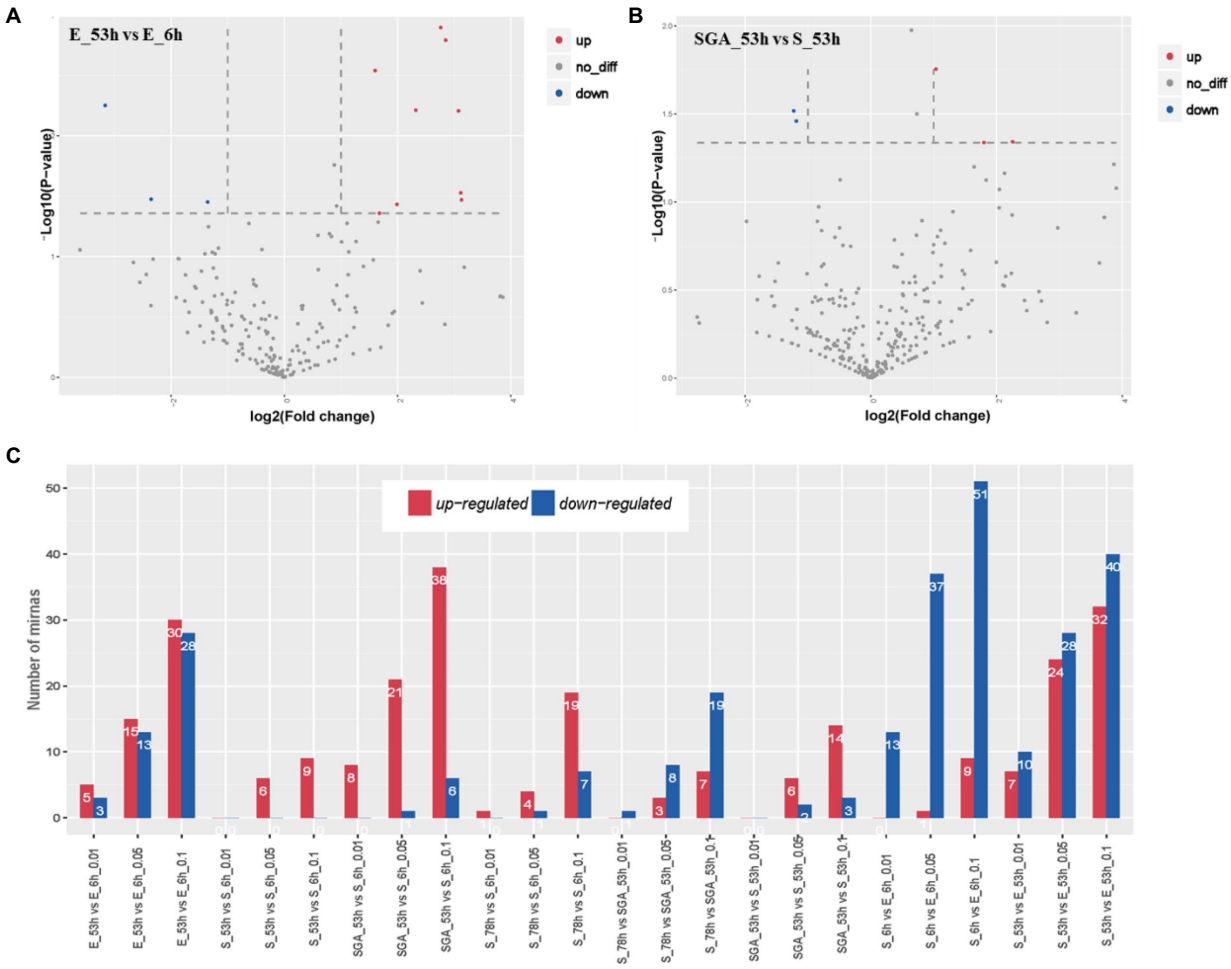
Differentially expressed miRNAs in different groups **(A)**, Volcano of differentially expressed miRNAs in the group of E_53h vs. E_6h. **(B)**, Volcano of differentially expressed miRNAs in the group of SGA_53h vs. S_53h. **(C)**, Differentially expressed miRNAs in different comparison groups.

To identify the miRNAs associated with maize seed vigor, we analyzed significant miRNAs among different samples (Figure 3). In Yu82, a maize inbred line with high germination rate, totally a number of 28 miRNAs with significant expression differences (*p* value ≦0.05) were discovered in the sample E_53h compared with that in the sample E_6h. More than half of the miRNAs were upregulated, suggesting that their complicated functions in the regulation of maize seed vigor. In Yu537A, a maize inbred line with low germination rate, a number of 6 miRNAs with significant expression differences (p value ≦ 0.05) were identified in sample S_53h compared with that in the sample S_6h. After GA3 treatment, the number of significantly differentially expressed miRNAs increased to 22, including 6 extremely significantly differentially expressed miRNAs, suggesting that more miRNAs were induced to be upregulated to involve in the regulation of maize seed vigor by GA3. From 53 h to 78 h after imbibition, hardly any noticeable changes were observed in Yu537A, which was significantly lower than that in the sample SGA_53h after GA3 treatment. Increased number of significantly differentially expressed miRNAs demonstrated that more complicated regulation mechanism in the sample of SGA_53h, which also indicated that GA3 played an important role in improving the vigor of maize seeds.

### Targets of miRNAs in seeds of two maize inbred lines

**Figure 4:**
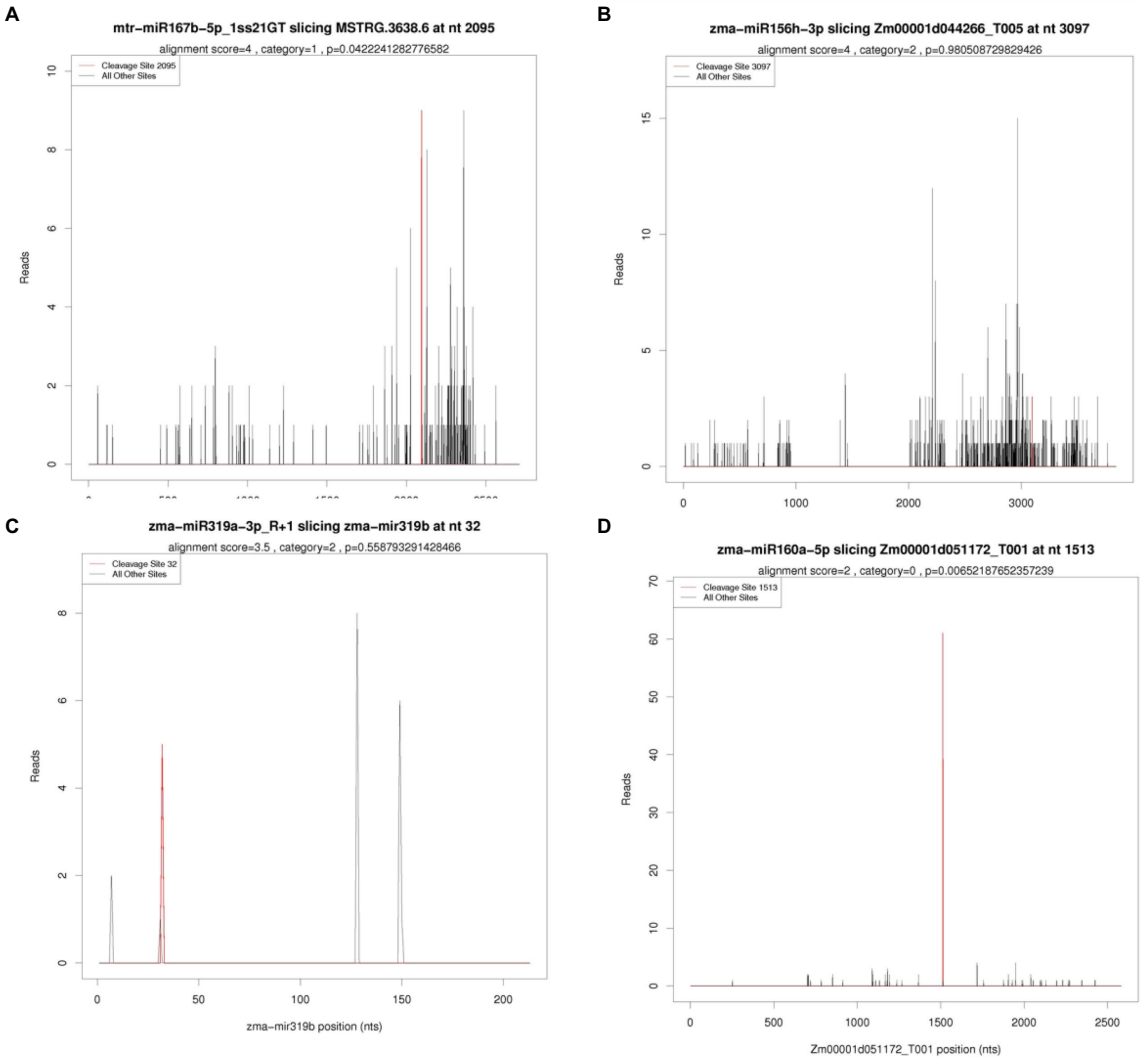
Typical categories of the target transcript according to the relative abundance of the tags at the target mRNA sites **(A–D)** represent T-plot results of different miRNAs. *X*-axis represent site information of mRNA sequence, while *Y*-axis represents the number of reads at a particular cutting site.

To understand the functions of identified miRNAs from seeds of two maize inbred lines, targets of identified miRNAs were predicted using recently developed degradome sequencing technology. In total, 24,186,228 (99.60%) mappable reads were obtained, including 6,559,673 unique mappable reads, while 20,126,668 (82.88%) of total reads were transcript mapped reads, including 4,692,740 unique transcript mappable reads, were obtained. By combing the targets result of miRNA prediction with the degradome sequencing, finally 6,196 targets (transcripts) were obtained (Figure 4; Supplementary Table 4). Among of which, the target number for one miRNA was varying from 1 to 998. Among of all targets identified, a total of 270 targets were identified in both degradome analysis and bioinformatics analysis (*p* value ≦ 0.05). Annotation and expression analysis of these targets (Supplementary Figure 1, 2) showed that the targets of miR160, miR167, miR319 and miR156 played an vital role at the regulation level of DNA transcription. Documents also indicated the targets of miR160, miR167, miR319 and miR156 were identified as transcription factors, such as SBP, ARE, MYB and TCB, respectively, which have been experimentally verified (Carrington and Ambros, 2003; Mallory and Vaucheret, 2006; Voinnet, 2009). This suggested the high reliability of target identification analysis in our research.

### Enrichment analysis of gene ontology and KEGG pathway of targets

**Figure 5:**
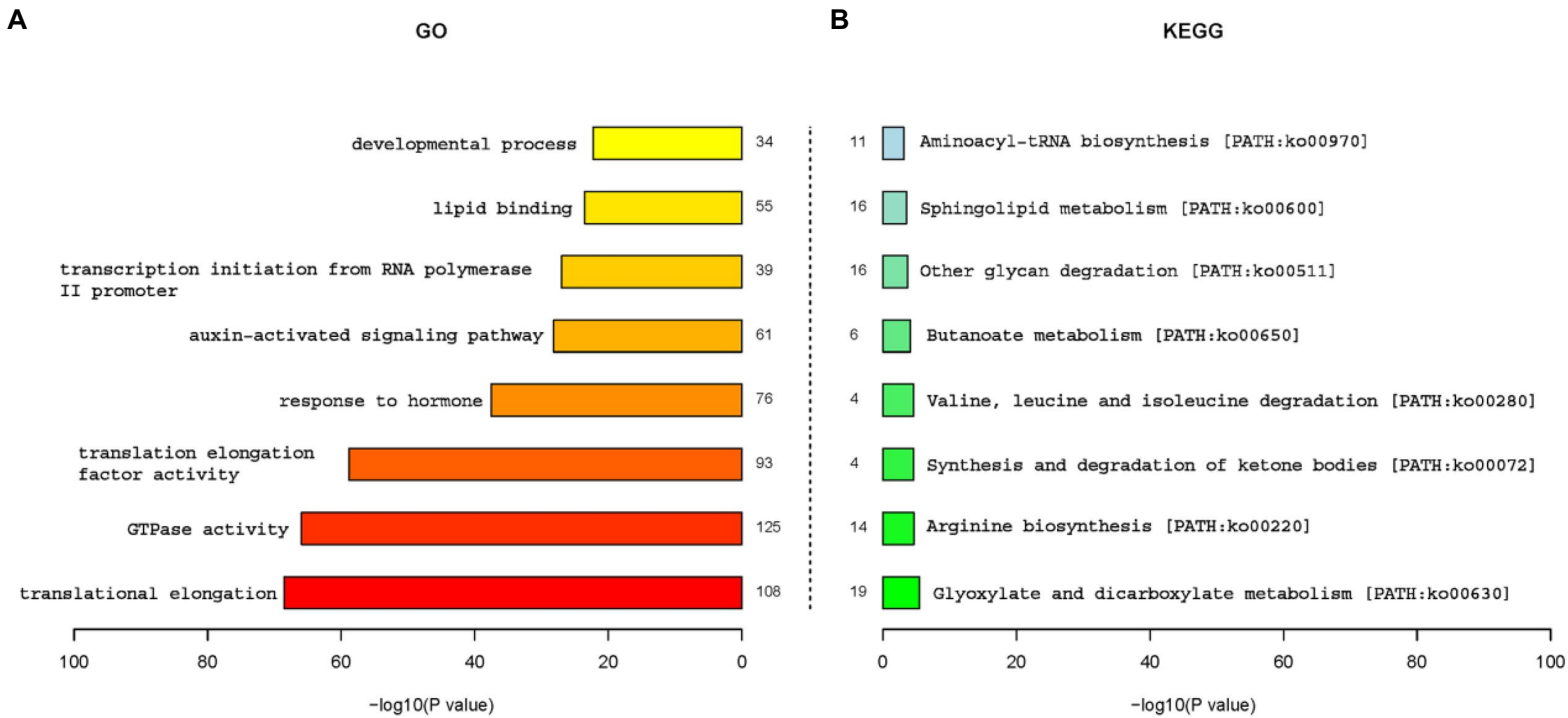
Gene ontology and functional classification of miRNA targets in two inbred lines. **(A)** Gene ontology analysis of miRNA targets. **(B)** KEGG analysis of miRNA targets.

To gain a better understanding of functional roles of miRNAs, GO and KEGG analysis of putative targets were conducted. All targets were annotated using GO annotations to describe the functions of genes and gene products, while KEGG analysis is used to understand the pathways of annotated targets. Figure 5 showed the GO and functional classification of miRNA targets in two maize inbred lines seeds. Obviously, target genes of translational elongation, GTPase activity, translation elongation factor activity and response to hormone were specially enriched by GO analysis (Figure 5A), which suggested that these genes may play an important role in the regulation of maize seed germination. KEGG analysis (Figure 5B) showed Glyoxylate and dicarboxylate metabolism [PATH: ko00630], Sphingolipid metabolism [PATH: ko00600], other glycan degradation [PATH: ko00511], and Arginine biosynthesis [PATH: ko00220] were several main pathways which were involved in the germination regulation of maize seeds.

### Function analysis of differentially expressed miRNAs and targets showed glycan degradation and galactose metabolism were closely correlated with improved maize seed vigor

**Figure 6:**
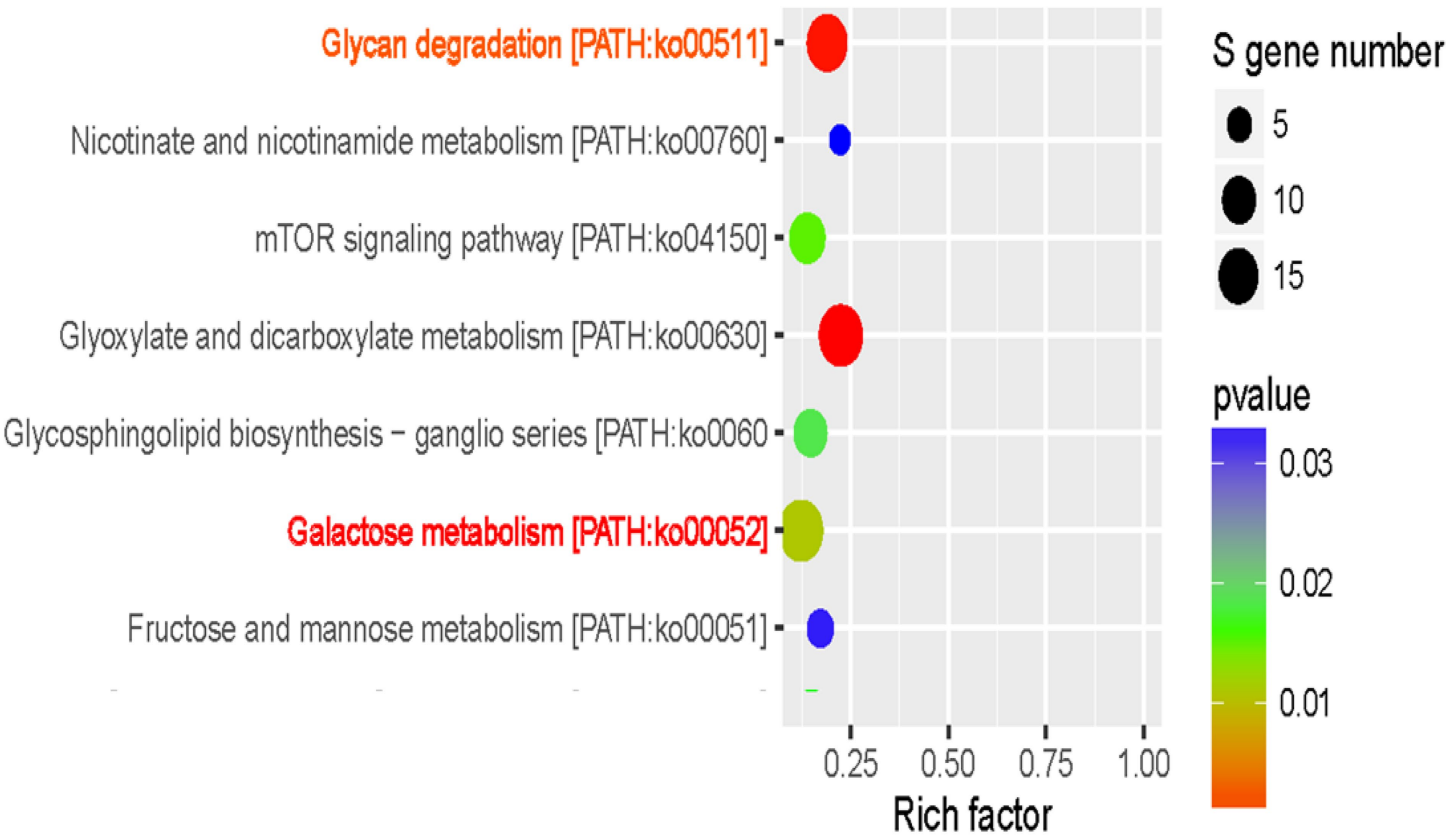
Kyoto Encyclopedia of Genes and Genomes enrichment analysis of targets.

**Figure 7:**
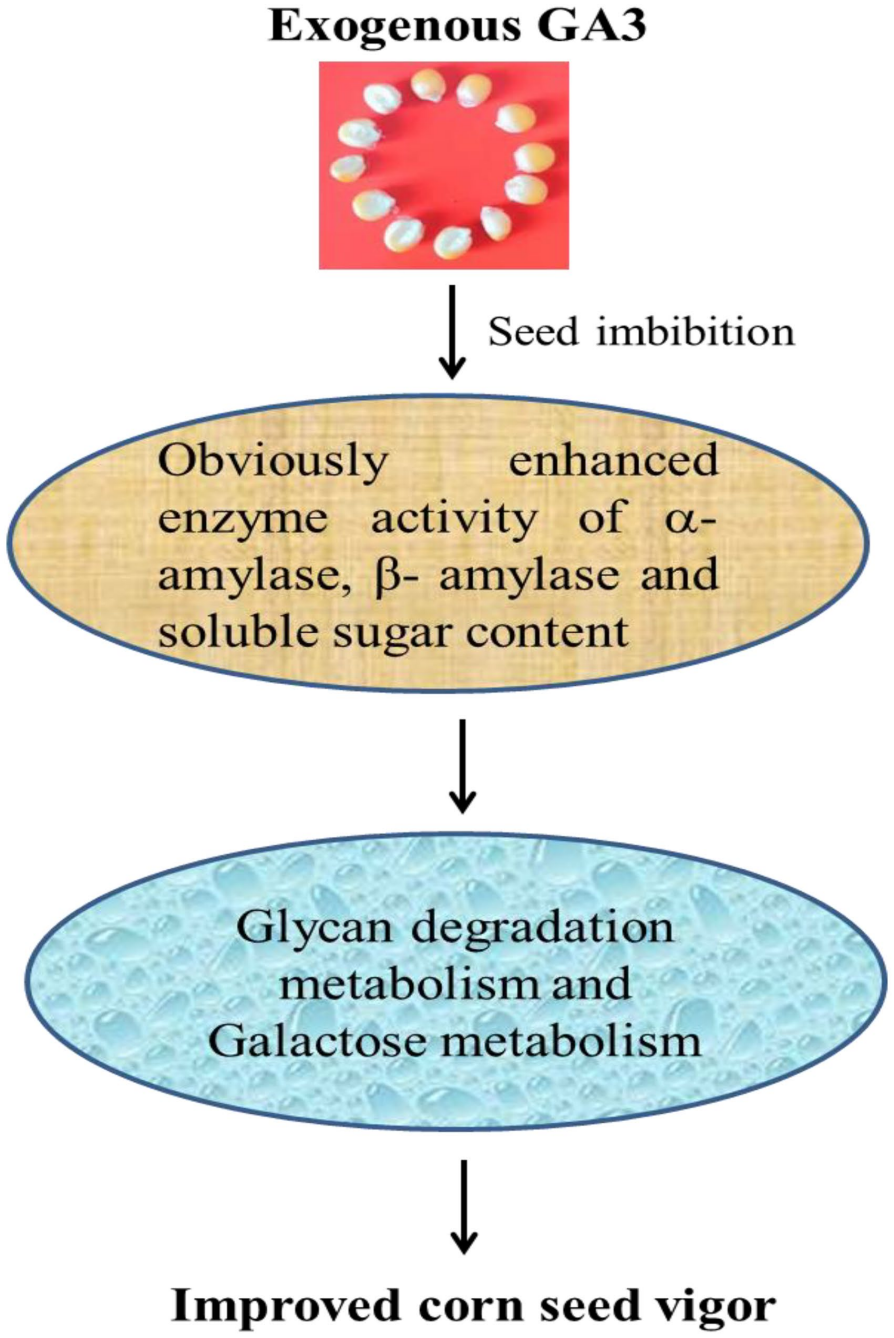
A regulation model for the improved maize seed vigor induced by GA3.

To decipher the complexity of regulatory networks closely related with the improved maize seed vigor, we further studied the significantly enriched pathways with the software ggplot2. Interestingly, two pathways named Glycan degradation [PATH: ko00511, *p* value = 0.0002] and Galactose metabolism [PATH: ko00052, *p* value = 0.01] were discovered and shown in Figure 6. A total of 16 miRNAs with different expressions were discovered in Glycan degradation while 19 differentially expressed miRNAs were obtained in Galactose metabolism. Various kinds of glycan are interconvertible in plants, and glycan can be hydrolyzed into small oligosaccharides and monosaccharides with reducing properties. At the same time, this could provide structural materials for the synthesis of new cells and organelles. So a regulation model was forecasted for the improved maize seed vigor induced by GA3 (Figure 7), which revealed main changes of glycans during maize seed germination, indicating that two metabolism pathways were closely correlated with improved maize seed vigor.

## Discussion

Seed germination is a so complex biological process regulated by a larger number of genes. Based on the whole germination process, GA3 treatment was proved to promote the germination rate of maize seeds significantly by breaking seed dormancy and inducing a series of related genes. In this study, two typical maize inbred lines were selected and used for the miRNAs and degradome sequencing analysis, containing Yu537A with low seed vigor and Yu82 with high seed vigor, which suggested that the materials were novel compared with previous published researches. Two maize inbred lines were derived from Yuzong5, which is an very popular variety and widely planted in China. So the background of two lines were similar, and comparative analysis of miRNA and degradome data could truly reflect the molecular mechanism difference between two inbred lines in the process of seed germination induced by gibberellin (GA3).

Seed vigor is a complex trait, including seed germination, seedling emergence and growth, as well as seed storability and stress tolerance, which is directly related with healthy plant seedlings (Zhao et al., 2021). In our research, six treatments were performed on two maize inbred lines to investigate the regulation mechanism of miRNAs in the germination process. After GA3 treatment, the number of differentially expressed miRNAs was significantly increased in the sample of SGA_53h, demonstrating that GA3 induced more complicated regulation mechanism to improve the vigor of maize seeds, which was not reported before (Zhang et al., 2009; Gong et al., 2015; Han et al., 2020). Seed dormancy, a common biological phenomenon, is an important biological process which was formed in the final stage of seed development, and this dormancy state would be conducive to seed vigor (Finch-Savage and Leubner-Metzger, 2006; Nelson et al., 2017). It is well known that plant hormones ABA and GA are a pair of hormone molecules that play hostile roles in the regulation of seed development (Lee et al., 2015), that is to say, ABA induces seed dormancy and inhibit seed germination while the function of GA was just the opposite (Li et al., 2021a). In our study, after GA3 treatment, the germination rate of two maize inbred lines was significantly improved, which is consistent with previous studies (Gong et al., 2015; Li et al., 2021a).

Analysis method of Multi-omics is often used to decode the molecular mechanism of various physiological activities in plants (Khan et al., 2015; Singer et al., 2021; Li et al., 2021b; Feng et al., 2022). Seed germination was a complicated process involving multiple genes and regulatory networks. After imbibition of two inbred seeds, seed germination needs the regulation of a large number of genes and the energy supply (Wolny et al., 2018). In this study, we performed miRNAs and degradome analysis, function analysis of differentially expressed miRNAs and targets showed Glycan degradation and galactose metabolism were closely correlated with improved maize seed vigor. Glycan degradation and galactose metabolism was also a kind of energy supply in the process of seed germination. Significantly increased α-amylase and β-amylase activity in our research suggested that polysaccharides in maize seeds were accelerated to be hydrolyzed, such as starch. Documents reported that sucrose and starch are used for Krebs cycle to produce ATP/NADPH and as structural substrates for the synthesis of DNA and cell walls (Li et al., 2021a). So it is speculated that increased enzyme activity of α-amylase and β-amylase induced by GA3 promoted the germination of maize seeds. Our results provided more evidence for energy supply during seed germination.

## Data availability statement

The original contributions presented in the study are publicly available. This data can be found at: https://www.ncbi.nlm.nih.gov/, GSE196738.

## Ethics statement

Permissions or licenses have been obtained to collect plant samples (two inbred lines Yu537A and Yu82). All methods about experimental research and field studies on plants were carried out in accordance with relevant guidelines and regulations.

## Author contributions

YJ and ZH designed the experiments and wrote the manuscript. BW and LT helped to carry out the experiments. LZ, SG, HZ, and LX managed the materials in the field and helped to perform the data analysis. All authors contributed to the article and approved the submitted version.

## Funding

This research was supported by grants from the National Natural Science Foundation of China (no. U1504315), Science and Technology Project in Henan Province (no. 212102110244), and the Scientific Research Foundation of Henan University of Science and Technology (no. 13480067).

## Conflict of interest

The authors declare that the research was conducted in the absence of any commercial or financial relationships that could be construed as a potential conflict of interest.

## Publisher’s note

All claims expressed in this article are solely those of the authors and do not necessarily represent those of their affiliated organizations, or those of the publisher, the editors and the reviewers. Any product that may be evaluated in this article, or claim that may be made by its manufacturer, is not guaranteed or endorsed by the publisher.
