# Supplementary figures and images for: Identification of miRNAs and their target genes associated with improved maize seed vigor induced by gibberellin
Source: Front Plant Sci. 2022 Sep 13;13:1008872. doi: 10.3389/fpls.2022.1008872 (PMC9514094; doi:10.3389/fpls.2022.1008872)

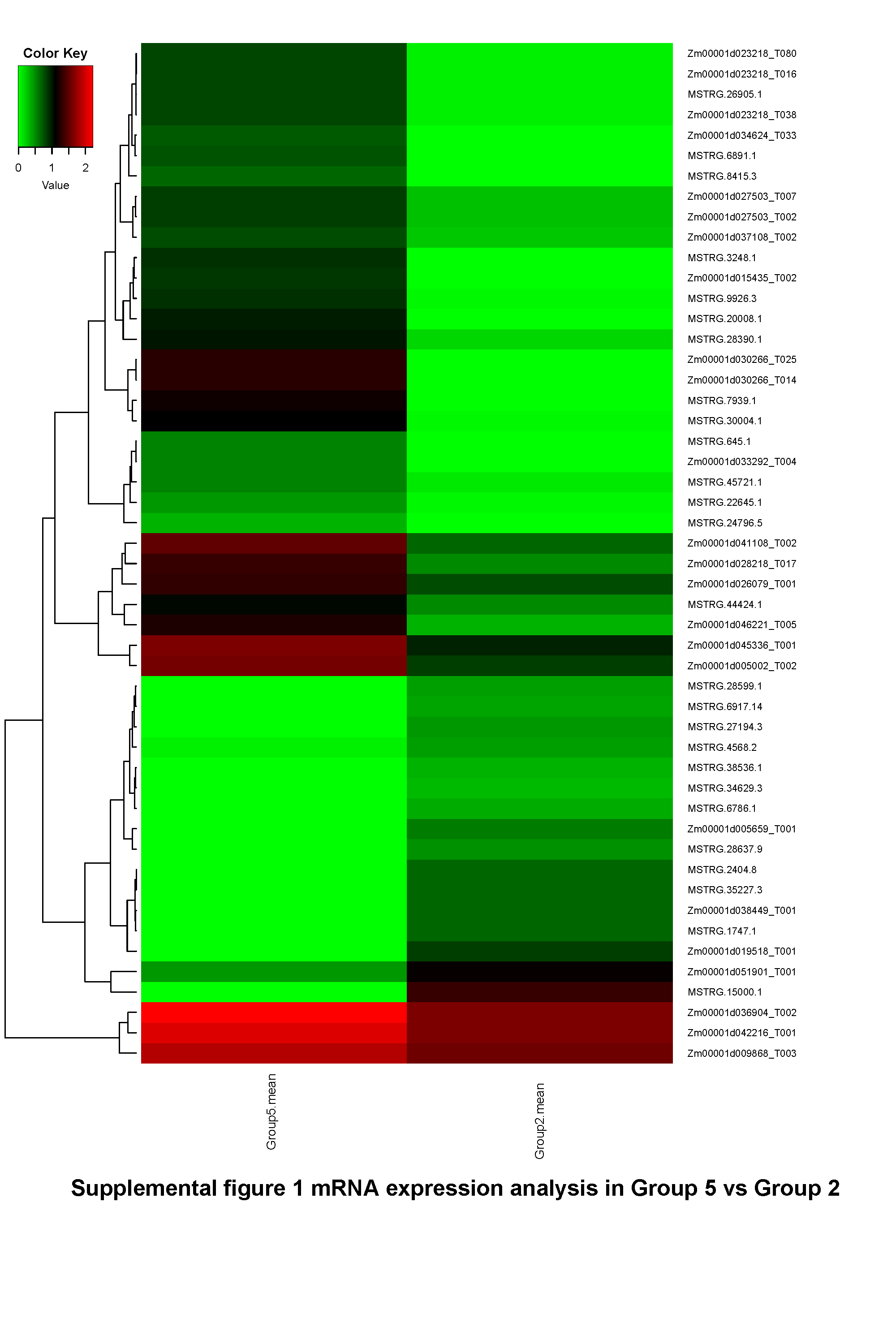

Supplement: Supplementary file 1 [file Image_1.PNG]

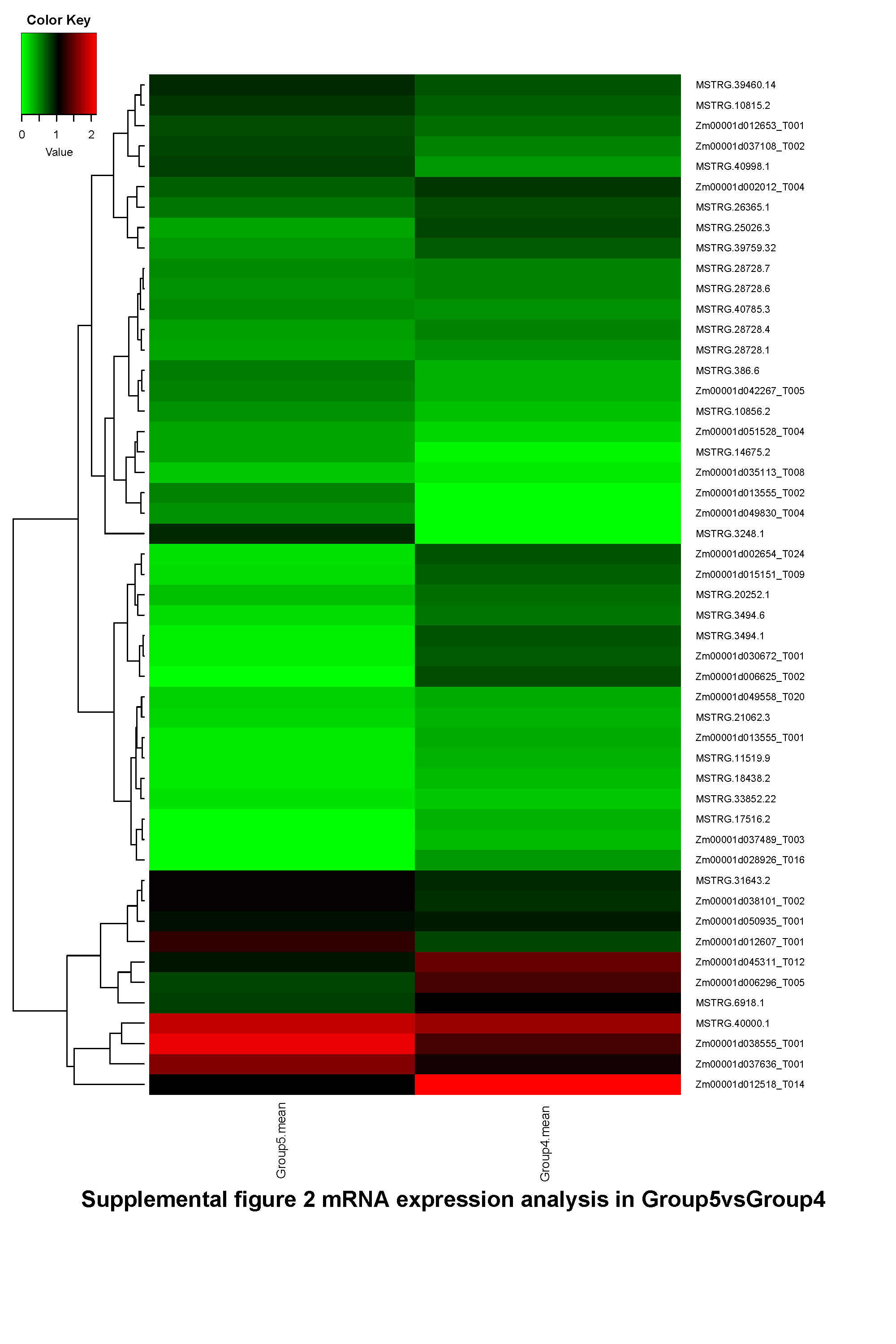

Supplement: Supplementary file 2 [file Image_2.PNG]
